# Length of Stay and Deaths in Diabetes-Related Preventable Hospitalizations Among Asian American, Pacific Islander, and White Older Adults on Medicare, Hawai‘i, December 2006–December 2010

**DOI:** 10.5888/pcd12.150092

**Published:** 2015-08-06

**Authors:** Mary W. Guo, Hyeong Jun Ahn, Deborah T. Juarez, Jill Miyamura, Tetine L. Sentell

**Affiliations:** Author Affiliations: Hyeong Jun Ahn, Biostatistics Core, John A. Burns School of Medicine, Honolulu, Hawai‘i; Deborah T. Juarez, Daniel K. Inouye College of Pharmacy, University of Hawai‘i at Hilo, Hilo, Hawai‘i; Jill Miyamura, Hawaii Health Information Corporation, Honolulu, Hawai‘i; Tetine L. Sentell, Office of Public Health Studies, University of Hawai‘i at Manoa, Honolulu, Hawai‘i.

## Abstract

**Introduction:**

The objective of this study was to compare in-hospital deaths and length of stays for diabetes-related preventable hospitalizations (D-RPHs) in Hawai‘i for Asian American, Pacific Islander, and white Medicare recipients aged 65 years or older.

**Methods:**

We considered all hospitalizations of older (>65 years) Japanese, Chinese, Native Hawaiians, Filipinos, and whites living in Hawai‘i with Medicare as the primary insurer from December 2006 through December 2010 (n = 127,079). We used *International Classification of Diseases – 9th Revision* (ICD-9) codes to identify D-RPHs as defined by the Agency for Healthcare Research and Quality. Length of stays and deaths during hospitalization were compared for Asian American and Pacific Islander versus whites in multivariable regression models, adjusting for age, sex, location of residence (Oahu, y/n), and comorbidity.

**Results:**

Among the group studied, 1,700 hospitalizations of 1,424 patients were D-RPHs. Native Hawaiians were significantly more likely to die during a D-RPH (odds ratio [OR], 3.92; 95% confidence interval [CI], 1.42–10.87) than whites. Filipinos had a significantly shorter length of stay (relative risk [RR], 0.77; 95% CI, 0.62–0.95) for D-RPH than whites. Among Native Hawaiians with a D-RPH, 59% were in the youngest age group (65–75 y) whereas only 6.3% were in the oldest (≥85 y). By contrast, 23.2% of Japanese were in the youngest age group, and 32.2% were in the oldest.

**Conclusion:**

This statewide study found significant differences in the clinical characteristics and outcomes of D-RPHs for Asian American and Pacific Islanders in Hawai‘i. Native Hawaiians were more likely to die during a D-RPH and were hospitalized at a younger age for a D-RPH than other studied racial/ethnic groups. Focused interventions targeting Native Hawaiians are needed to avoid these outcomes.

## Introduction

Diabetes rates are higher among many Asian American and Pacific Islander populations than among whites (1). Native Hawaiians have some of the highest diabetes rates in the world (2,3). Diabetes risk increases considerably with age. Medicare covers most diabetes care for participants aged 65 years and older. The Asian American and Pacific Islander population in the United States is approximately 12 million and is increasing rapidly (4); therefore, the numbers of Medicare beneficiaries who are Asian Americans or Pacific Islanders are likely to grow faster than many other racial/ethnic groups (5).

Hospitalizations are an important area of focus in diabetes care because they account for a major portion of health care cost (6,7) and are highly burdensome to individuals and families. Diabetes-related potentially preventable hospitalizations (D-RPH) are of particular concern and were the target of recent policy action (8,9). Potentially preventable hospitalizations are defined by the Agency for Healthcare Research and Quality (AHRQ) as hospitalizations preventable with accessible and effective outpatient disease management (9).

Among Hawaiian residents aged 65 years or older, Native Hawaiians and some Asian groups are significantly more likely to have D-RPHs, even after considering the high diabetes prevalence rates in these racial/ethnic groups (10). However, little is known about the outcomes of these hospitalizations. The goal of this study was to compare deaths and length of stay for D-RPH in Hawai‘i for major Asian American and Pacific Islander subgroups and for older (≥65y) whites with Medicare coverage. These 2 outcomes were chosen because they are regularly used, can be compared with findings from other locations, and are relevant to patients, providers, and health care administrators (11–14). Death is a measured outcome of primary importance (11), and length of stay is a commonly reported indicator for hospitalizations. Longer hospitalizations are more expensive, are burdensome to patients, and can put patients at greater risk for hospital-associated adverse events (12,13). Among older adults, length of stay is a particularly important issue because many older patients experience functional and cognitive declines during hospitalization (14).

Hawai‘i provides excellent opportunities to research D-RPHs. Hawai‘i has a large population of older adults (15), growing rates of diabetes (16), and a diverse ethnic population. Almost 40% of Hawai‘i’s population is Asian American (primarily Filipino, Chinese, and Japanese), and approximately 10% are Native Hawaiian or other Pacific Islanders (17). As such, hospital data in Hawai‘i have unique details about Asian American and Pacific Islander racial/ethnic groups not captured in state-level data in most other locations, which allows for disaggregated data on heterogeneous Asian and Pacific Islander groups.

## Methods

The Hawaii Health Information Corporation (HHIC) is an independent, not-for-profit health care data organization that collects and validates detailed inpatient discharge data at the patient level from all hospitalizations by all insurers in the state. HHIC data include information on race/ethnicity of patients, insurer, age, sex, and *International Classification of Diseases – 9th Revision – Clinical Modification* (ICD-9) codes (18). Patients in long-term care or psychiatric hospitals are excluded. HHIC data are used as the Hawai‘i hospital data source for the major national inpatient database (19), an indication of their high quality. For our sample, we considered nonpregnancy-related hospitalizations from December 2006 through December 2010, of Hawaiian residents aged 65 years or older with Medicare as the primary payer (n = 144,253). Hospitalizations were excluded if they lacked valid race/ethnicity data (n = 2,706) or they did not report Japanese, Chinese, Native Hawaiian, Filipino, or white as their racial/ethnic group (n = 14,468). These 5 racial/ethnic groups are the major racial/ethnic groups in Hawai‘i. After exclusions, there were a total of 127,079 eligible hospitalizations.

### Sample

Following AHRQ definitions, we used ICD-9 discharge codes to identify D-RPH (20). We included in our sample patients hospitalized with 1) uncontrolled diabetes without mention of a short-term or long-term complication, 2) diabetes with short-term complications (eg, ketoacidosis, hyperosmolarity, coma), 3) diabetes with long-term complications (eg, renal, eye, neurological, circulatory complications; complications not otherwise specified), and 4) lower-extremity diabetes-related amputations. More detail is available from AHRQ (http://www.qualityindicators.ahrq.gov/Downloads/Modules/PQI/V31/pqi_guide_v31.pdf). Although people with a potentially preventable hospitalization for short-term or long-term diabetes care may have had uncontrolled diabetes in the past, there is no overlap by AHRQ definitions of preventable hospitalizations for uncontrolled diabetes and potentially preventable hospitalizations for short-term and long-term diabetes complications.

### Variables

The HHIC race/ethnicity variable is created from the categories available consistently across all hospitals in Hawai‘i from December 2006 to December 2010 (18). One primary race is reported across all hospitals, typically from patient self-report at intake. Mixed-race individuals are represented as their self-reported primary race. The 5 included racial/ethnic groups were identified consistently across all Hawai‘i hospitals during the study period.

The dependent variable, death during stay, was identified from the hospital record’s discharge disposition as “expired.” Length of stay was measured by the total number of days of each potentially preventable hospitalization. We controlled for sex on the basis of administrative data and for 3 age categories (65–74 y, 75–84 y, and ≥85 y). We also controlled for comorbidities as measured by the Charlson Comorbidity Index (CCI) (21). The CCI is a commonly used measure suitable for hospital discharge data that sums the total 17 potential comorbidities (eg, heart failure, dementia, lymphoma) weighted by severity into a single comorbidity score (21). A higher score indicates more comorbidity, which is associated with a greater likelihood of death and higher use of hospital resources. Location of residence (lives on Oahu versus does not live on Oahu) was also controlled.

### Statistical analyses

Death during stay and length of stay were compared across Asian American and Pacific Islander subgroups and whites in unadjusted analyses and analyses were adjusted for age, sex, location of residence, and comorbidity. After identifying patients across all hospitals using the HHIC data, we included only 1 hospitalization per patient for the analyses of in-hospital death. From these unique patients, we compared the likelihood of in-hospital death during a preventable hospitalization by race/ethnicity by using χ^2 ^and a multivariable logistic model. Length of stay was compared by race/ethnicity by using all hospitalizations rather than unique patients. A repeated measure, negative binomial regression model, was used to address potential correlation within patients in these analyses as these were not restricted to one hospitalization per patient.

## Results

We found 1,700 D-RPHs during the study period by 1,424 unique patients. Significant differences were seen across racial/ethnic groups for many clinical characteristics (Table 1). D-RPHs by Asian Americans and Pacific Islanders had significantly higher percentages of female patients than D-RPHs by whites. The majority of patients lived on Oahu; this percentage ranged from 56% for Native Hawaiians to 94% for Chinese. Notably, 59% of Native Hawaiians with a D-RPH were in the youngest age group (65–75 y), whereas only 6.3% were in the oldest age group (≥85 y). By contrast, 23.2% of Japanese patients were in the youngest age group, and 32.4% of them were 85 years or older.

**Table 1:** Demographic Characteristics of Medicare Recipients Aged 65 Years or Older (n = 1,424) With Diabetes-Related Preventable Hospitalizations (n = 1,700), Hawai‘i, December 2006–December 2010

Characteristic	Chinese	Filipino	Native Hawaiian	Japanese	White	*P* Value^a^
Number of patients	98	282	254	518	272	NA
Number of hospitalizations	116	341	308	599	336	NA
Hospitalizations per patient, mean (SD)	1.18 (0.48)	1.21 (0.55)	1.21 (0.55)	1.16 (0.50)	1.23 (0.61)	.35
**Age at last diabetes-related preventable hospitalization, y, n (%)**						
65–74	22 (22.5)	101 (35.8)	151 (59.4)	120 (23.2)	127 (46.7)	<.001
75–84	51 (52.0)	139 (49.3)	87 (34.2)	230 (44.4)	101 (37.1)	<.001
≥85	25 (25.5)	42 (14.9)	16 (6.3)	168 (32.4)	44 (16.2)	<.001
Female	48 (49.0)	164 (58.4)	140 (55.1)	267 (51.4)	118 (43.2)	.007
Living on Oahu	92 (93.9)	216 (76.6)	141 (55.5)	409 (79.0)	162 (60.0)	<.001
Expired	b	b	18 (7.1)	16 (3.1)	b	.017
Length of stay (days), mean (SD)	9.1 (11.1)	7.4 (8.8)	10.9 (16.8)	8.6 (11.9)	10.2 (14.1)	.015
Charlson Comorbidity Index, mean (SD)	4.8 (3.1)	5.0 (3.2)	6.4 (3.6)	4.4 (3.1)	5.4 (3.1)	<.001

Abbreviation: SD, standard deviation; NA, not applicable.

a The *P* value for “expired” for race/ethnicity was derived by using a χ^2^ analysis. The *P* value for length of stay by race/ethnicity was derived by using an analysis of variance.

b The value of this cell is less than 10 and cannot be presented because of data privacy rules regarding number of patients less than 10.

In unadjusted outcome variables, Native Hawaiians with a D-RPH had the longest average lengths of stay, followed by whites and Chinese. There were 53 deaths total in our study group. Seven percent of D-RPH among Native Hawaiians ended in death compared with 3.1% of D-RPHs among Japanese. D-RPHs by Native Hawaiians also had the highest comorbidity scores, with the average CCI score 2 points higher than the CCI scores for Japanese D-RPHs, 1.8 points higher than for Chinese D-RPHs, and 1.4 points higher than for Filipino D-RPHs.

Multivariable models show predictions of length of stay and death during stay by race/ethnicity after adjusting for comorbidity, age, sex, and living on Oahu (Table 2). Compared with whites, Native Hawaiians were significantly more likely to die during a D-RPH (OR, 3.92; 95% CI, 1.42–10.87). Filipinos had significantly shorter length of stay (RR, 0.77; 95% CI, 0.62–0.95) than whites. No other racial/ethnic differences were significant in multivariable models. In the model predicting in-hospital mortality, no control factors were significant. In the model predicting longer length of stay, other significant factors were being aged 64 to 74 years, being male, and having more comorbid conditions.

**Table 2:** Results From Multivariable Models Predicting Length of Stay and Death Among Medicare Patients Aged 65 years or Older (n = 1,424) With a Diabetes-Related Preventable Hospitalization (n = 1,700), Hawai‘i, December 2006–December 2010^a^

Demographic Characteristic	Deaths, Odds Ratio (95% CI)	Length of Stay, Relative Risk (95% CI)
**Race/ethnicity**		
Chinese	2.71 (0.75–9.81)	0.90 (0.69–1.17)
Filipino	1.76 (0.58–5.38)	0.77 (0.62–0.95)
Native Hawaiian	3.92 (1.42–10.87)	0.96 (0.75–1.22)
Japanese	1.69 (0.60–4.76)	0.90 (0.74–1.09)
White	Reference	Reference
**Age**		
65–74	0.86 (0.39–1.92)	1.20 (1.01–1.42)
75–84	0.74 (0.34–1.60)	1.11 (0.94–1.32)
≥85	Reference	Reference
**Female versus male**	0.96 (0.55–1.69)	0.81 (0.70–0.93)
**Living on Oahu (yes vs no)**	1.40 (0.72–2.72)	1.05 (0.89–1.23)
**Charlson Comorbidity Index (1 unit increase)**	1.07 (0.98–1.16)	1.10 (1.08–1.11)

Abbreviation: HHIC, Hawaii Health Information Corporation; CI, confidence interval.

a Taken from Hawaii Health Information Corporation data (18).

To provide insight into our study findings, we also considered the frequencies of specific D-RPHs by racial/ethnic group (Figure). The most common D-RPHs were long-term complications, followed by amputations. Fewer D-RPHs were for short-term complications and uncontrolled diabetes. Native Hawaiians, in particular, had few D-RPHs for short-term complications and uncontrolled diabetes and high percentages for amputations.

**Figure:**
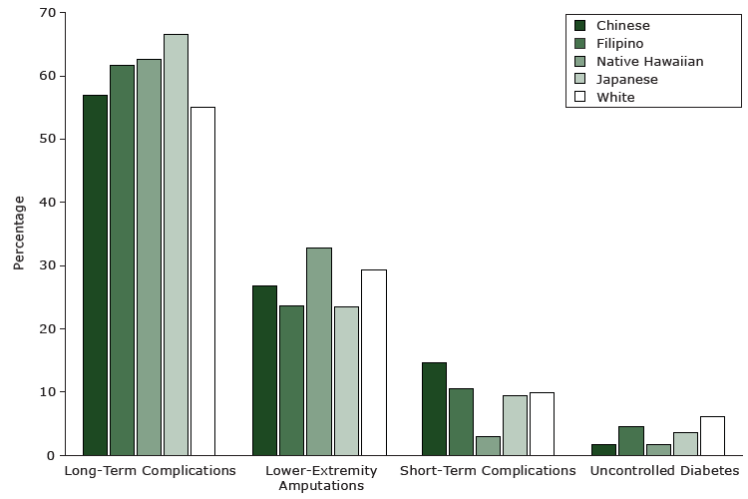
The percentages of types of diabetes-related preventable hospitalizations in Hawai‘i from December 2006 through December 2010, by racial/ethnic group. Data are from the Hawaii Health Information Corporation (18). All comparisons by race/ethnicity within type are significant at *P* < .05. **Table:** 

Reason for Hospitalization	Chinese	Filipino	Native Hawaiian	Japanese	White
Long-term complications	56.9	61.6	62.7	66.6	55.1
Lower-extremity amputations	26.7	23.5	32.8	23.4	29.2
Short-term complications	14.7	10.6	2.9	9.5	9.8
Uncontrolled diabetes	1.7	4.4	1.6	3.5	6.0

## Discussion

This statewide study found significant differences in the clinical characteristics and outcomes of D-RPHs for Asian American and Pacific Islander groups. Disparities were seen particularly for Native Hawaiians, who were more likely to die during a D-RPH and were hospitalized at younger ages for a D-RPH than other studied racial/ethnic groups. D-RPHs of Native Hawaiians were more likely to involve an amputation, which was the most expensive of the 4 types of D-RPHs encountered in previous research (22).

A primary value of this research is to demonstrate the depth and impact of diabetes-related disparities for Native Hawaiians. Previous studies have found that Native Hawaiians aged 65 years or older are not only diagnosed with diabetes at higher rates than other populations in Hawai‘i (23) but are also hospitalized at higher rates for preventable diabetes-related conditions than whites, even after adjusting for the higher diabetes prevalence in this population (10). Furthermore, this study finds that Native Hawaiians covered by Medicare are more likely to die during these preventable hospitalizations. This finding suggests that even after controlling for comorbidity as we do in this study, Native Hawaiian older adults are going to the hospital sicker than other studied racial/ethnic groups. Clinicians both inside and outside the hospital should be aware of this disparity, because it suggests a greater urgency for treatment in this population. Primary care clinicians may need to refer older Native Hawaiian patients to treatment earlier than patients in other racial/ethnic groups. Future research should determine whether Native Hawaiians are sicker on admission to a hospital than other racial/ethnic groups because of delayed access to care and devise innovative solutions (eg, greater use of peer educators) (24,) to ensure that even vulnerable members of these populations are receiving timely access to health care.

Our findings may also be useful for community health centers and other organizations providing comprehensive, preventive services to help underscore the importance of strong chronic disease care management. Patients may also be interested in knowing of the apparent inequity in Native Hawaiian deaths during D-RPHs to underscore the importance of both condition management and promptly seeking treatment when an urgent condition (eg, sign of infection) arises.

A previous study found that older Japanese, Filipino, and Chinese adult populations were also more likely to be hospitalized for a D-RPH (even considering the higher diabetes prevalence in these groups) than older white adults (10). However, in this study, these racial/ethnic groups did not have a greater likelihood of poorer D-RPH hospitalization outcomes than whites. This suggests that for older Asians, unlike for older Native Hawaiians, the disparities for D-RPHs compared with whites end at the point of higher rates of these hospitalizations. Native Hawaiians, however, are more likely to die during a D-RPH. This finding provides a better portrait of the issues and disparities for diabetes-related outcomes among older Asian and Native Hawaiian populations. Better understanding of the patterns of D-RPHs can guide more focused interventions for understudied groups of patients.

Our findings of health care disparities among Native Hawaiian D-RPHs are of interest because our study focuses on residents of Hawai‘i covered by Medicare. A uniform payer, such as Medicare, could potentially reduce disparities in outcomes. Other evidence suggests that even as a uniform payer, Medicare does not resolve disparities between Asian Americans and Pacific Islanders and whites in quality of care (5). For instance, 1 study showed that Asian Medicare beneficiaries had longer waits to see a doctor and were less likely to receive diabetes-related eye examinations or be tested for hemoglobin A1c, lipid levels, and microalbuminuria, or to receive self-care instruction (5). Yet Medicare, which begins coverage at age 65 or at the point of disability, cannot fully resolve disparities that arise from many years of poorly controlled diabetes before a person becomes eligible for Medicare. Ideally, the poor diabetes-related outcomes seen in this study, particularly among Native Hawaiians, could be resolved by preventing, or better controlling, diabetes at young ages. For this reason, our study findings should be of interest to Medicaid and to private insurers who are the primary payers of working-age people. This study also underscores the importance of existing, innovative programs seeking to reduce diabetes prevalence across diverse Asian American and Pacific Islander groups, including the PILI ‘Ohana Project (25) and the Diabetes Detection and Prevention Project (26).

We did not find significantly longer lengths of stay for D-RPHs among Asian and Pacific Islander groups than among whites. Instead, we that found Filipinos had significantly shorter lengths of stay for D-RPHs. Longer lengths of stay may signify more complications or greater danger for hospital-associated adverse events. On the other hand, shorter lengths of stay may be associated with more adverse events following discharge (13). This would be an informative area of research among Filipinos. Another area for future research is determining whether the shorter length of diabetes-related hospital stays among Filipinos is due to less severe illness, distinct comorbidities compared to other racial/ethnic groups, or other factors. The CCI factors evaluated in this study did not indicate that Filipinos had fewer comorbidities than other racial/ethnic groups.

We also found differences in clinical characteristics across groups that could inform the design and focus of interventions. Even among an older population, Native Hawaiians experienced a D-RPH at a younger age than other racial/ethnic groups studied. These findings are consistent with previous research (10) and are particularly troubling because they are coupled with the greater likelihood that Native Hawaiians will die during preventable hospitalizations.

Although this study has numerous strengths, including 4 years of data from a large, statewide sample and representation of diverse Asian American and Pacific Islander groups, the study has some limitations. These data do not provide information on many factors that might be useful to explain or better understand health disparities among racial/ethnic groups, particularly some clinical factors such as depression and social determinants such as familial support. We were unable to consider factors such as obesity, time since diagnosis, education, or proximity to primary care (27–29). We were also unable to include other Pacific Islanders (eg, Samoans, Tongans, Micronesians), either generally or as specific subgroups, in our analyses because the sample sizes of these groups were too small to perform comparative, multivariate analyses. These areas require further study, especially given that other Pacific Islanders have known health disparities in diabetes and are extremely understudied (30,31). Another limitation is that possible differences in coding across hospitals may have affected our analysis (32). Findings may also reflect preferences for the location of end-of-life care or differences in access to information about options for end-of-life care. Some racial/ethnic groups may be more likely to be transferred to hospice than others. It is also possible that the relatively small number of deaths during a D-RPH may have affected our ability to find significant differences in this outcome.

Efforts of the Centers for Medicare and Medicaid Services to address disparities have not traditionally focused on Asian Americans and Pacific Islanders (5). Yet estimates suggest that by 2050, approximately 1 of every 10 Americans, approximately 38 million, will be of Asian or Pacific Islander descent (4). Evidence from this study suggests that Medicare should pay particular attention to disparities among older Native Hawaiians, because members of this group were more likely to die during a D-RPH than older white adults and were more likely to experience a D-RPH at a younger age than other racial/ethnic groups studied. Focused interventions are needed that target Native Hawaiians to avoid these outcomes. Further study is also needed to determine how the mix of diagnoses and heavier burden of comorbidities might contribute to the higher in-hospital mortality rate for this population.
